# Fast and Sensitive Screening of Oxandrolone and Its Major Metabolite 17-Epi-Oxandrolone in Human Urine by UHPLC—MS/MS with On-Line SPE Sample Pretreatment

**DOI:** 10.3390/molecules26020480

**Published:** 2021-01-18

**Authors:** Jaroslav Galba, Juraj Piešťanský, Andrej Kováč, Dominika Olešová, Ondrej Cehlár, Martin Kertys, Petr Kozlík, Petra Chaľová, Barbora Tirčová, Kristián Slíž, Peter Mikuš

**Affiliations:** 1 Department of Pharmaceutical Analysis and Nuclear Pharmacy, Faculty of Pharmacy, Comenius University in Bratislava, Odbojarov 10, 832 32 Bratislava, Slovakia; galba@fpharm.uniba.sk (J.G.); piestansky@fpharm.uniba.sk (J.P.); petra.chalova@gmail.com (P.C.); kristian.sliz@uniba.sk (K.S.); 2 Biomedical Research Center of the Slovak Academy of Sciences in Bratislava, 84510 Bratislava, Slovakia; 3 Toxicological and Antidoping Center, Faculty of Pharmacy, Comenius University in Bratislava, Odbojarov 10, 832 32 Bratislava, Slovakia; 4 Institute of Neuroimmunology, Slovak Academy of Sciences, Dubravska cesta 9, 84510 Bratislava, Slovakia; andrej.kovac@savba.sk (A.K.); dominika.olesova@savba.sk (D.O.); ondrej.cehlar@savba.sk (O.C.); 5 Department of Pharmacology, Jessenius Faculty of Medicine in Martin, Comenius University in Bratislava, 036 01 Martin, Slovakia; martin.kertys@uniba.sk; 6 Biomedical Center Martin, Jessenius Faculty of Medicine in Martin, Comenius University in Bratislava, 036 01 Martin, Slovakia; 7 Department of Analytical Chemistry, Faculty of Science, Charles University, Hlavova 8, 128 43 Prague 2, Czech Republic; petr.kozlik@natur.cuni.cz; 8 Department of Chemistry, Faculty of Natural Science, Matej Bel University in Banska Bystrica, 974 09 Banska Bystrica, Slovakia; barbora.tircova@umb.sk

**Keywords:** oxandrolone, 17-epi-oxandrolone, human urine, ultra-high performance liquid chromatography, tandem mass spectrometry, on-line SPE extraction

## Abstract

Oxandrolone, a synthetic testosterone analog, is used for the treatment of several diseases associated with weight loss. Unfortunately, oxandrolone is abused by many athletes and bodybuilders due to its strong anabolic effect. We have developed and validated a highly sensitive and rapid on-line SPE-UHPLC-MS/MS method for the determination of oxandrolone and simultaneous identification of its major metabolite 17-epi-oxandrolone in urine matrices. Enrichment of the analytes via an integrated solid-phase extraction was achieved using an Acquity UPLC BEH C18 Column. Subsequently, the chromatographic separation of the on-line preconcentrated sample fraction was achieved using an Acquity HSS T3 C18 Column. For the structural identification of these analytes, a high-resolution mass spectrometer Synapt-G2Si coupled to the Acquity M-class nano-LC system with ionKey source was used. A highly sensitive determination of oxandrolone was achieved using a tandem quadrupole mass spectrometer XEVO TQD. The method was successfully validated in the linear range of oxandrolone from 81.63 pg·mL^−1^ (limit of quantification, LOQ) to 5000 pg·mL^−1^ in the human urine matrix. It was applied to the analysis of real urine samples obtained from a healthy volunteer after the oral administration of one dose (10 mg) of oxandrolone. Concentration vs. time dependence was tested in the time interval of 4 h–12 days (after oral administration) to demonstrate the ability of the method to detect the renal elimination of oxandrolone from the human body. Favorable performance parameters along with successful application indicate the usefulness of the proposed method for its routine use in antidoping control labs.

## 1. Introduction

**Figure 1:**
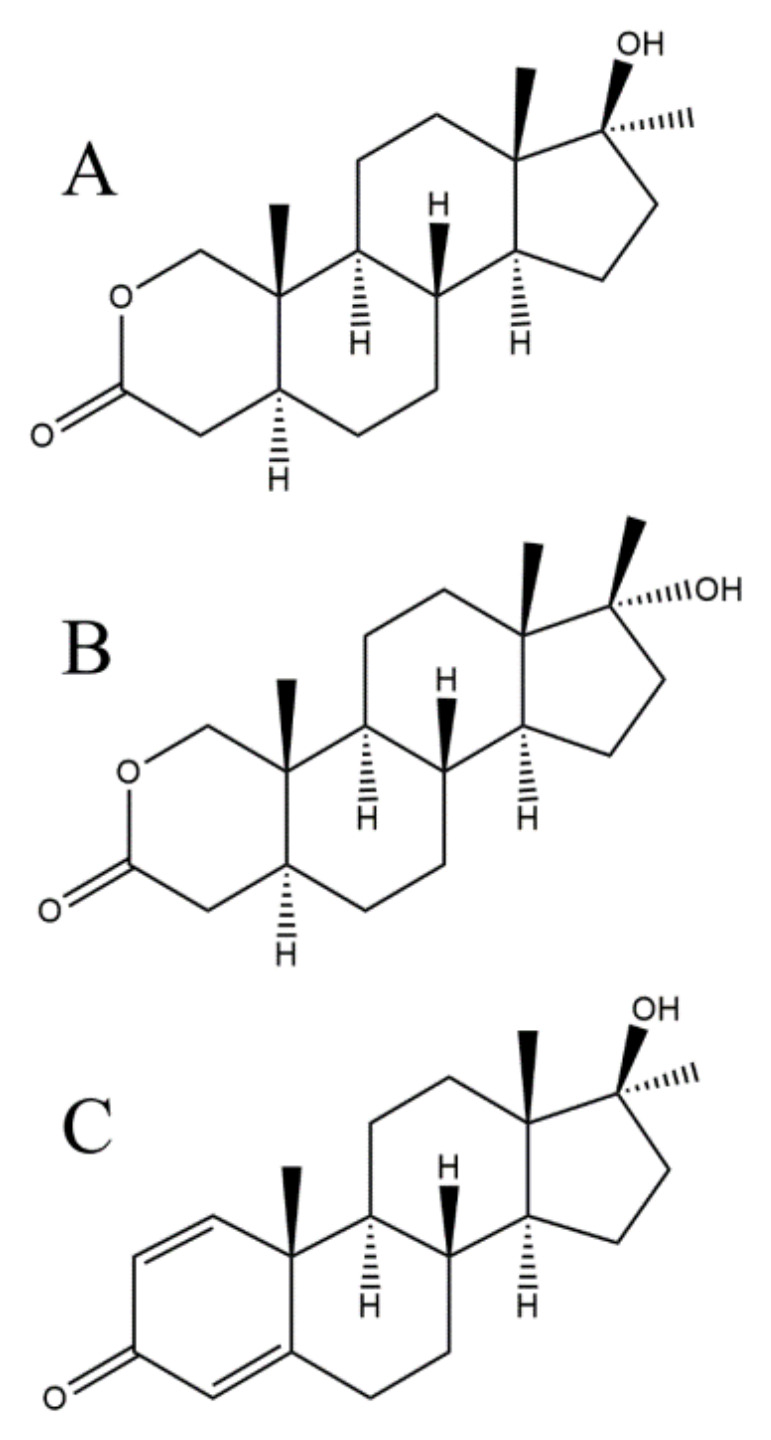
Chemical structure of (**A**) oxandrolone, (**B**) epi-oxandrolone, and (**C**) the methandienone as IS.

Oxandrolone (5a-androstan-2-oxa-17a-methyl-17b-ol-one), OXA, is a synthetic testosterone analog (Figure 1A) synthesized in 1962 [1]. Testosterone and its analogs are anabolic-androgenic steroids (AASs) [2] exerting effects both anabolic and androgenic by binding to the androgen receptor (AR). The androgenic effects cover the development and maintenance of secondary sexual characteristics while the anabolic effect means protein synthesis promotion and skeletal muscle growth [3]. The addition of an alkyl group to the C17 of OXA improves its oral bioavailability profile and allows it to be orally administered [2]. This is a major advantage of OXA compared with other anabolic steroids. The Food and Drug Administration (FDA) approves its use as a weight gain adjunct following major trauma, extensive surgery [4], reversing muscle catabolism in cachectic and alcoholic hepatitis [5,6,7], HIV/AIDS [2,8,9], and in patients with burns [10,11,12]. OXA has been used for the treatment of Turner syndrome [13] and Klinefelter syndrome [14,15]. Various AASs have different ratios of androgenic and anabolic activity depending on the extent of binding affinity to ARs in various tissues. OXA is highly anabolic with only a few androgenic effects in the ratio anabolic:androgenic effects 10:1 [16,17]. OXA is abused by many athletes and bodybuilders due to its strong anabolic effect and it is especially suitable for use in women owing to weak androgenic effects [18,19].

The first study of OXA metabolism in humans was described by Schänzer [20,21] using gas chromatography quadrupole mass spectrometry. It was shown that OXA was mainly excreted in an unchanged form and that its 17α-epimer (Figure 1B) is the major metabolite. These results were confirmed by the counter synthesis in 1993 [18,21]. Gas chromatography coupled with a quadrupole or triple quadrupole mass analyzer with electron ionization is still routinely used for the determination of OXA in urine in doping control [18,22,23,24,25]. Revelsky at al. [26] used a low-resolution TOF mass spectrometer coupled with gas chromatography for the determination of OXA after derivatization with N,O-bis(trimethylsilyl)trifluoroacetamide (BSTFA) as a reagent. The GC-MS method showed a good specificity, unfortunately, it required time-consuming sample preparation consisting of several steps. Moreover, the GC analysis run time took tens of minutes.

Several HPLC-MS methods have been developed for the monitoring of OXA and its metabolites in human urine [27]. Leinonen et al. developed LC-MS/MS methods for the detection of OXA and epi-oxandrolone in human urine using different types of ionization, namely electrospray ionization (ESI), atmospheric pressure chemical ionization (APCI), and atmospheric pressure photoionization (APPI) [28,29]. Viryus, et al. [30] coupled high-resolution mass spectrometry (HRMS) (orbitrap) with HPLC via APCI for screening doping controls. The same group developed an HPLC-HRMS method enabling identification of OXA in the urine as long as two weeks after ending its 15-day administration [31]. Guddat, et al. [32] used GC-MS/MS and LC-MS/MS for the identification of OXA and its metabolites in urine samples. The most frequently employed sample clean-up procedure in these LC-MS analyses of urine samples containing OXA was liquid-liquid extraction (LLE). It was applied after sample hydrolysis and followed by evaporation of organic solvent and reconstitution by a mobile phase [27,29,30,31,32]. The solid-phase extraction (SPE), as sample preparation for an LC-MS/MS analysis of oxandrolone sulfate conjugates in urine, was described in 2016 by Rzeppa and Viet [33]. When performing off-line, however, both sample preparation approaches suffered from several time-consuming steps requiring relatively large sample volumes (from 1 to 5 mL for LLE and 0.5 mL for SPE), and still were not sufficient to obtain LOQ values below 1 ng·mL^−1^.

The aim of this work was to develop and validate an advanced UHPLC-MS/MS method with on-line SPE extraction, expecting to provide an enhanced effectivity and performance parameters for identification and quantification of OXA and its major metabolite 17-epi-oxandrolone in multicomponent matrices. The applicability of such a method for a study of renal OXA elimination and, by that, a common antidoping control, was verified via the analysis of urine samples taken from a healthy volunteer after peroral administration of one dose of OXA.

## 2. Results and Discussion

### 2.1. Liquid Chromatography—Quadrupole-Time-of-Flight (LC-QTOF) Method

**Figure 2:**
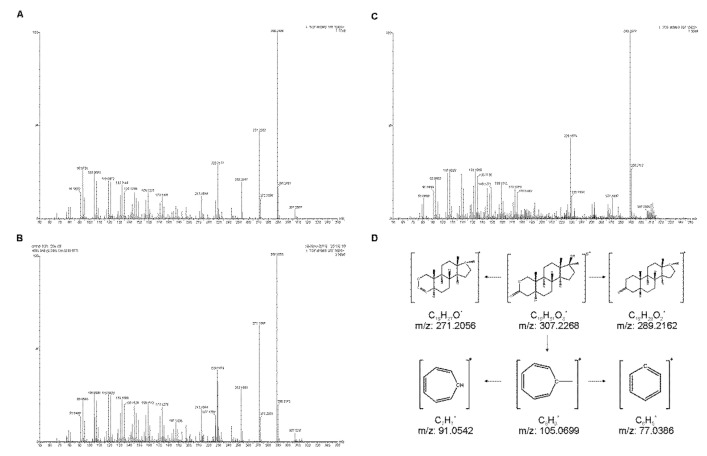
LC-QTOF identification of oxandrolone (OXA) and its metabolite. The MS2 spectrum of (**A**) standard OXA, (**B**) OXA in a urine sample, (**C**) OXA metabolite 17-epi-oxandrolone in a urine sample, and (**D**) the structures of molecular and daughters ions used for identification and quantification.

Applying the LC-QTOF method and conditions (Section 2.2), the molecular weight of OXA was measured with an excellent mass accuracy as 307.2127 (theoretical monoisotopic mass 307.2121) with the mass error below 1 ppm even in the samples containing complex urine matrices. In the tandem mass spectrum of standard OXA (Figure 2A), the dominant daughter ion species was detected at m/z 289.2496 (not used for quantification due to nonspecific water losses in a collision cell); 271.2332 (used for quantification 307.3 → 271.2); 229.2177 (identifier), and 93.0791 (identifier). These were further used as daughter ions in the triple quadrupole detection (see following sections). The use of m/z = 271.2 as a quantifier in the triple quadrupole detection was selected according to the fact that the most intensive daughter ion (m/z = 289.3) represented a relatively nonspecific fragmentation ion (loss of water). The selection of this quantification transition is supported by the fact that many steroids have a very similar fragmentation pattern. This represents a serious issue that is compounded, if, for reasons of convenience or increased sensitivity, a relatively nonspecific daughter ion is monitored (e.g., a “water loss”). Fragmentations that include such common losses should be avoided wherever possible as these can be nonspecific for the target analytes.

The MS2 spectrum of OXA from a urine sample obtained after oral administration of one OXA dose is shown in Figure 2B. The presence of OXA metabolite, 17-epi-oxandrolone, was confirmed in the same sample via the exact mass of the molecular ion, isotope ratio, and MS2 (Figure 2C). These findings were in good agreement with the previous published papers which deal with the analysis of OXA and its isobaric metabolite 17-epi-oxandrolone [27,29]. MS2 spectrum for both timely resolved compounds (retention time of OXA was 5.22 min and retention time of 17-epi-oxandrolone was 5.70 min) contained daughter ions 105.0681 and 93.0693 that are characteristic for this steroid structure [27]. The chemical structures of molecular and daughter ions used for identification and quantification are in Figure 2D.

### 2.2. SPE-UHPLC-MS/MS (QQQ) Method Optimization

*MS optimization.* Optimization of the MS detection step was performed with the use of OXA standard solution at the 1000 ng·mL^−1^ concentration level. The following parameters were optimized in the MS detection stage: cone voltage (tested range 2–100 V), desolvation gas flow (tested range 500–1000 L·h^−1^), desolvation gas temperature (tested range 200–400 °C), and capillary voltage (tested range 1–3 kV). The results obtained during the MS conditions optimization are clearly summarized in Table S1 (Supplementary Material). The optimum values of these parameters were 28 V, 700 L·h^−1^, 400 °C, and 3 kV, respectively, considering the highest stability and intensity of the analytical signal as the main criteria. The dependence of the peak area (extracted traces for OXA) on the collision energy was tested in the range of 2–80 V. Optimum values of the collision energy for the produced OXA fragments, providing their highest peak areas, are summarized in Table 1. The selected MS conditions were used in a further optimization procedure of the separation (UHPLC) and sample preparation (SPE) step.

*UHPLC optimization.* Optimization of the UHPLC separation step was performed with the use of OXA standard solution at the 1000 ng·mL^−1^ concentration level. The first step in the optimization of UHPLC separation was the selection of a proper stationary phase. Several reverse phase columns with different interaction mechanisms were tested: (i) ethylene-bridged hybrid inorganic-organic particle (BEH) containing columns with reverse stationary phases (Acquity BEH C18, Acquity BEH C8, and Acquity BEH Shield C18 with BEH by incorporating an embedded carbamate group into the bonded phase ligand), (ii) silica-based columns with improved retention of polar compounds (Acquity HSS T3, Acquity HSS Cyano), and (iii) Acquity CSH C18 based on (BEH) particle technology column with a charged surface. The mobile phase consisted of acetonitrile and 0.1% formic acid in MPW. The flow rate was 0.4 mLmin^−1^, temperature 40 °C. The tested amount of acetonitrile in the mobile phase was from 40% to 90% (isocratic elution). The dependences of the elution times of OXA standard (1000 ng·mL^−1^) on the percentual amount of acetonitrile for various stationary phases are summarized in Table S2 (Supplementary Materials). The increased amount of acetonitrile was responsible for shortening of the retention time. Contrarily, the separation efficiency (expressed as number of theoretical plates—N, and corrected for the unretained peak retention time) was decreasing with the increase of acetonitrile in mobile phase. The best peak shape, peak area, analytical signal intensity of OXA and appropriate separation efficiency were achieved using the Acquity HSS T3 (Waters Corporation, Milford, MA, USA) column as a stationary phase and a 50% amount of acetonitrile in the mobile phase. In the next step, two mobile phase buffers with different concentrations of ammonium formate (10 mM and 20 mM in 0.1% FA) were compared with 0.1% FA. The signal of the OXA standard was decreased by about 30% and 45% when using the ammonium formate buffers, compared with 0.1% FA (Table S3; Supplementary Materials). Besides better sensitivity, the optimum mobile phase composition (50% acetonitrile with 0.1% FA) provided also an enhancement in the column efficiency, see data in Table S3 (Supplementary Materials).

**Figure 3:**
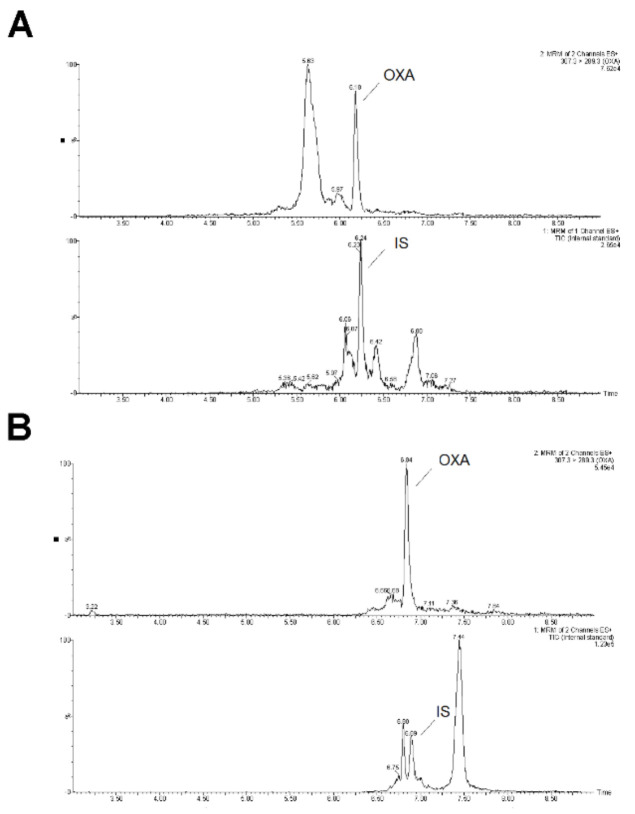
Elimination of urine matrix interferents in the on-line SPE sample pretreatment. Influence of the loading time of the washing solution (30% CAN) on the sample clean-up. MRM chromatogram of oxandrolone (OXA) and methandienone as IS: (**A**) urine spiked with the standard of OXA and IS with the loading time of the washing solution to be 1.5 min, (**B**) urine spiked with the standard of OXA and IS with the loading time of the washing solution to be 3.0 min.

**Figure 4:**
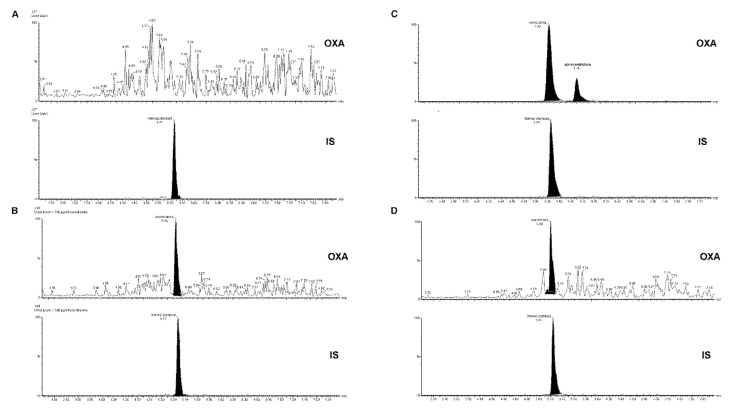
Representative chromatograms of oxandrolone (OXA) and methandienone as IS illustrating a real biomedical application of the SPE-UHPLC-MS/MS method. (**A**) Blank urine spiked with IS; (**B**) blank urine spiked with standard OXA (100 pg·mL^−1^) and IS, (**C**) urine sample taken 48 h after administration of one tablet (Oxandrix (DMX laboratories), 10 mg declared content per tablet) (OXA, Rt = 5.22 min; 17-epi-OXA, Rt = 5.70 min), (**D**) urine sample taken 9 days after the administration (OXA, Rt = 5.20 min). MRM transition 307.3 → 271.2 for OXA and 17-epi-oxandrolone, and 301.3 → 149.2 for IS were used.

*SPE optimization.* In the sample preparation step, the following extraction columns were tested for the optimization of on-line SPE analyte enrichment: Xbridge C18 2.1 × 30 mm with 10 µm particles (Waters), Xbridge C8 2.1 × 30 mm (10 µm), Oasis HLB 2.1 × 30 mm (10 µm), and Acquity BEH C18 2.1 × 50 mm (1.7 µm) (Waters). The concentration of acetonitrile in the loading solution was tested from 10% to 40%. At first the OXA standard (1000 ng·mL^−1^) was loaded with various injection volumes (10–200 µL). As an optimum, the Acquity BEH C18 2.1 × 50 mm column was chosen for the on-line SPE enrichment, providing the highest extraction recovery in the injection volumes ranging from 100 µL to 200 µL. No significant differences in the recovery were registered in the concentration interval of acetonitrile 10–30%, while for 40% ACN the recovery was zero (i.e., OXA was completely eluted from the SPE column to the waste without any enrichment). A 30% ACN concentration was chosen as an optimum, with respect to the maximum removal of possible organic interfering urine matrix constituents during OXA enrichment. These SPE conditions were approved with the use of urine matrix spiked with the OXA standard (final OXA concentration in urine sample was 1000 ng·mL^−1^). The data obtained during the SPE optimization are clearly summarized in Table S4 (Supplementary Material). Additional optimization of the sample clean-up in the on-line SPE step (performed with the use of spiked urine matrix) was based on changing the loading time of the washing solution to 30% ACN (to elute the urine matrix constituents), see Figure 3. By changing the loading time in the SPE from 1.5 min to 3 min we partially succeeded in suppressing the interference while maintaining the recovery (compare panels A and B in Figure 3). Subsequently, the effect of pH and ionic strength of the loading solution on a urine matrix interference removal was tested. The solutions of ammonium formate (10, 20, and 40 mM) at different pH values (6.2, 5.5, and 4.5) were considered. A 10 mM formate at pH 6.2 was selected as the optimum loading solution enabling the removal of the highest amount of potentially interfering compounds from the matrix, and thereby, creating favorable conditions for the practical use of the developed SPE-UHPLC-MS/MS method (Figure 4A,B).

### 2.3. SPE-UHPLC-MS/MS (QQQ) Method Validation

**Figure 5:**
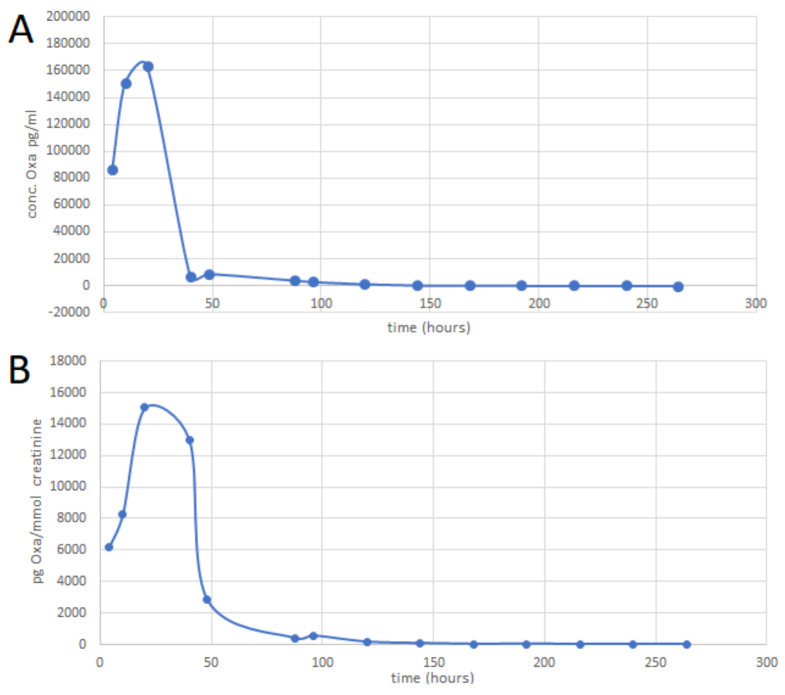
Time vs. concentration profiles of OXA eliminated from the body in urine. OXA was monitored in the urine samples taken from a healthy volunteer after administration of a 10 mg dose of OXA (one tablet Oxandrix). Each point in the profiles corresponds to a mean obtained from 3 consecutive measurements. (**A**) Time vs. concentration profile of OXA, (**B**) time vs. concentration profile of OXA employing quantitative OXA data normalized to creatinine.

Validation of the screening method was performed in accordance with the FDA guidelines [34]. Calibration dependence was examined by measuring the calibration standards of OXA in the concentration range of 20–5000 pg·mL^−1^. The calibration curve with an equation of y = 0.000779x + 0.01517 (Figure S1; Supplementary Materials) was linear and measured with acceptable precision and accuracy in the range of 81.63 (LOQ)–5000 pg·mL^−1^. Linearity parameters, the standard deviation of the intercept and slope, the limit of quantification, limit of detection, number of theoretical plates, and height equivalent to one theoretical plate are shown in Table 2. These data indicated suitable linearity, concentration range, and sensitivity for practical biomedical use of the proposed method. For the illustration, Figure 5 shows chromatograms of blank urine (panel A) and spiked urine (panel B) at a concentration level of OXA close to its LOQ (100 pg·mL^−1^) obtained by the SPE-UHPLC-MS/MS method.

The data obtained from the analysis of QC samples are summarized in Table 3. The accuracy for OXA at three concentration levels ranged from 93.4 to 109.5%. The intraday and interday precisions for OXA were below 11.0%. The obtained data accomplished the FDA criteria for acceptable accuracy (±15% of nominal concentration) and precision (±15% RSD) of the method, and, by that, supported its usefulness for practical use.

Additional performance parameters brought similar positive conclusions for the developed method. The recovery, calculated from the QC samples at three concentration levels, was higher than 88% (Table 4). The values of the matrix effect using the IS were below 16% (Table 4). The stability of OXA samples under different conditions (i.e., dwelling in autosampler, freeze-thaw) was acceptable as it is indicated by data given in Table S5 (Supplementary Material). The changes in quantitative parameters of the analyte undergoing the tested processes were below 15%.

### 2.4. SPE-UHPLC-MS/MS (QQQ) Method Application

The optimized and successfully validated SPE-UHPLC-MS/MS method was applied to monitor OXA and its main metabolite epi-oxandrolone in the urine samples taken from a healthy volunteer after administration of 10 mg dose of OXA (one tablet Oxandrix).

As the very first step, the presence of glucuronide and sulfate metabolic forms of OXA in the real samples was examined in order to unify possible OXA forms for the best analyte recovery. For this purpose, the urine samples were hydrolyzed enzymatically using β-glucuronidase, which provides both β-glucuronidase and arylsulfatase activity [35], according to the procedure described previously [31]. There were found no significant differences in OXA concentrations between the hydrolyzed samples and the samples prepared according to the procedure described in Section 3.4.3. (Table S6) (Supplementary Material). This indicated no glucuronide and sulfate metabolic forms of OXA in the volunteer’s urine samples and, by that, no necessity for enzymatic hydrolysis as an additional sample preparation step. Hence, the simple sample preparation procedure described in Section 3.4.3 was applied for all biomedical experiments.

Representative analytical profiles obtained from the SPE-UHPLC-MS/MS analysis of the volunteer´s urine samples are shown in Figure 4 (panels C and D). The MRM chromatograms of OXA and its metabolite epi-oxandrolone in the urine taken 48 h and 9 days after the drug administration are depicted in panels C and D, respectively. Maximum levels of OXA in urine were detected till 20 h after the drug administration. The amount of OXA in the urine on the 9th day was close to the LOQ value while the OXA concentrations corresponding with later sample collections were below the LOD. The epi-oxandrolone metabolite was detectable 7 days after the drug administration with the maximum levels detected in urine between 20–40 h after the administration. Time vs. concentration profiles of OXA eliminated from the body in urine is depicted in Figure 5. Basic and normalized (to creatinine) data of OXA and the ratios between OXA and epi-oxandrolone peak-areas are summarized in Table S7 (Supplementary Material).

The above stated and discussed results highlighted the analytical and application potential of the developed SPE-UHPLC-MS/MS method, and its usefulness for a reliable, fast, and sensitive monitoring of OXA and its main metabolite epi-oxandrolone in real human urine samples. When briefly comparing the present method with relevant published methods, only a few articles reported the determination of OXA in urine matrices to study time vs. concentration dependences. Guddat et al. [32] analyzed OXA and its long-term metabolites, but OXA (unlike its long-term metabolites) could not be detected more than ca. 3 days after drug administration. Viryus et al. [31] were able to detect OXA in urine by high-resolution mass spectrometry up to 14 days after ingestion, but the volunteers were taking a dose of 10 and 20 mg per day during the 15 days before sample collection. Moreover, the developed SPE-UHPLC-MS/MS method is characterized by a possibility to identify and detect very low concentrations of oxandrolone in comparison to another previous published method [33]. The LOD value investigated by our method was ~40-times better in comparison to the LOD declared by Rzeppa et al. [33]—24.5 pg·mL^−1^ vs. 1000 pg·mL^−1^. This is also beneficial in terms of WADA technical document requirements [36,37]. The application range of the proposed 2D approach could be further spread (even in 2–3 orders and fmol·L^−1^ LOQ levels) when using more sensitive MS (e.g., newer QQQ). Anyway, the developed 2D LC-MS method principally provides better possibilities for sensitive and fast monitoring of OXA and its metabolite in urine matrices than conventional (1D) LC-MS methods, and a higher degree of automatization of the whole analytical process (including on-line sample preparation) makes this advanced 2D approach favorable for routine use.

## 3. Materials and Methods

### 3.1. Chemicals and Reagents

Acetonitrile (LC-MS grade), β-glucuronidase Type HP-2 from Helix pomatia 100,000 units/mL was obtained from Sigma-Aldrich (Steinheim, Germany). Ammonium formate and formic acid were purchased from Fluka (Chemika, Switzerland). OXA and Methandienone (Figure 1C), serving as an internal standard (IS), were obtained as reference substances from Dr.Ehrenstorfer (Augsburg, Germany). High-purity water (MPW) was prepared by a Millipore Direct Q water purification system obtained from Merck (Darmstadt, Germany). OXA tablets were obtained from an internet shop (https://dmx-labs.com/en/) as Oxandrix (10 mg) from DMX laboratories. The amount of the active substance (OXA) in the tablets was determined by an LC-MS/MS method according to the previously published paper [38].

### 3.2. LC-QTOF Instrumentation and Conditions

The high-resolution mass spectrometry analysis was performed using a Synapt-G2Si instrument coupled to an Acquity M-class nano-LC system and equipped with an ionKey source (Waters Corporation, Milford, MA, USA). A separation device (chip) iKey Peptide BEH (100Å, 18 µm, 150 µm × 100 mm) was used for separation. The iKey device was heated to 40 °C. Mobile phase A was composed of formic acid in MPW (0.1%, *v*/*v*), and mobile phase B consisted of acetonitrile with 0.1% formic acid (*v*/*v*). A mobile phase gradient program was as follows: 10% B (0–1 min), increasing to 90% B (1–15 min) and then returning to 10% B and re-equilibrating at 17.1 to 20 min. The flow rate was 3 µL·min^−1^ and the injection volume was 3 µL. In a mass spectrometer, nitrogen was used as a drying gas and high purity nitrogen (N2) was used as a collision gas for collision-induced dissociation (CID). The mass spectrometric parameters were as follows: positive ion mode; desolvation gas flow 800 L·h^−1^; desolvation temperature 350 °C; capillary voltage 2.8 kV; source temperature 100 °C; mass range, *m*/*z* 50–1200 for MS1.

### 3.3. Online SPE-UHPLC-QQQ Instrumentation and Conditions

A Waters Acquity UPLC I-Class System was used in this study and configured with a sample manager, a column thermostat with two 2 position/6 port switching valves, and a binary solvent manager. Column eluates were detected with a triple-quadrupole mass spectrometry detector (XEVO TQD) through an electrospray ionization source. Data were acquired and processed by Mass Lynx software (all Waters Corporation, Milford, MA, USA).

**Figure 6:**
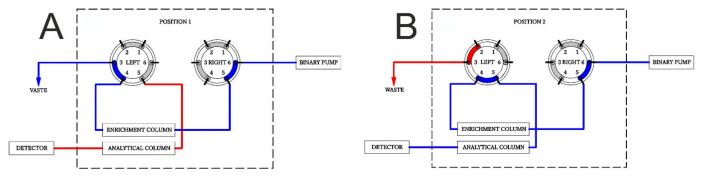
Scheme of SPE enrichment process in SPE-UHPLC-MS/MS method. Diagrams illustrating two positions of switching valve: (**A**)—load position, (**B**)—elute position. Enrichment column: Acquity UPLC BEH C18 Column (1.7 µm, 2.1 × 50 mm) (Waters), loading solution: ammonium formate (10 mM; pH = 6.2)/ACN (30%), elution solution: ammonium formate (10 mmol/L; pH = 6.2)/ACN (30–90%) gradient.

The analyte enrichment on the on-line SPE was achieved through an Acquity UPLC BEH C18 column (1.7 µm, 2.1 × 50 mm) (Waters) equipped with a column filter (on-line filter, 0.22 μm) (Waters) in front of the column. The chromatographic separation was achieved with an Acquity HSS T3 C18 Column (1.7 μm, 2.1 × 50 mm) (Waters). The column for SPE enrichment was maintained at 20 °C. The temperature of the analytical column was 40 °C. The temperature of the sampler was 10 °C and the injection volume was 200 μL. The positions of the switching valves were: 0.00 min, left valve—position 1, 2.50 min left valve—position 2, and 10.00 min left valve to position 1. The right valve was constantly in position 2. The connections among the ports in both positions are displayed in Figure 6. Mobile phase A consisted of ammonium formate (10 mM, pH = 6.2) in water. Mobile phase B consisted of 100% acetonitrile. The elution started at 30% B (0–2.6 min), increasing to 90% B (2.6–5.5 min), returning to 30% B (8.9–9.0 min), and re-equilibrating (9.0–11.0 min) before the next injection. The flow of the loading pump (binary pump) was 0.4 mL·min^−1^. Optimized parameters in the SPE enrichment process are summarized in Supplementary Material, Table S8.

A positive electrospray ionization mode (ESI+) was used in the ESI-MS stage. The main operational parameters of the mass detector were as follows: the source block temperature was 150 °C, the capillary voltage was 3000 V, desolvation gas flow rate was 700 L·h^−1^, desolvation temperature was 400 °C, cone gas flow rate was 50 L·h^−1^. A dwell time for each transition was 44 ms. Ions of the analyte and internal standard were monitored in the selected reaction-monitoring (SRM) mode. The MS parameters and conditions for OXA and methandienone are summarized in Table 1.

### 3.4. Sample Preparation

#### 3.4.1. Standard Solutions

The stock solutions of OXA (100 µg·mL^−1^) and Methandienone (IS) (100 µg·mL^−1^) were prepared separately by dissolving 1 mg of OXA reference standard and 1 mg of the methandienone reference standard in 10 mL of a water solution of methanol (50:50 *v*/*v*), and it was stored at −20 °C in the freezer. The stock solution of OXA was serially diluted with a 50% ACN/MPW to obtain working standard solutions of the desired concentration range (from 2 ng·mL^−1^ to 1000 ng·mL^−1^). The IS stock solution was diluted with the 50% ACN/MPW to give a concentration of 200 ng·mL^−1^ (working solution). The calibration standards were prepared by adding 20 µL of the working solutions of OXA into 1970 μL blank urine samples (4 times diluted with 40% ACN/MPW) and with the internal standard working solution (10 μL, 200 ng·mL^−1^). The tested calibration concentrations of OXA ranged from 20 to 5000 pg·mL^−1^.

#### 3.4.2. QC Samples

The quality control (QC) samples were prepared from blank urine samples (4 times diluted with 40% ACN/MPW) spiked with the working solution of OXA at low, medium, and high concentrations (for the recovery and matrix effect testing 75, 750, and 5000 pg·mL^−1^, respectively, and for the intra- and interday accuracy and precision testing 100, 250, and 1000 pg·mL^−1^, respectively) and with the working solution of IS (10 μL, 200 ng·mL^−1^).

#### 3.4.3. Urine Samples

A 500 μL volume of the urine sample, 1490 μL of 40% ACN/MPW, and 10 μL of IS solution (200 ng·mL^−1^) were mixed. The mixture was vortexed for 20 s and then centrifuged at 30,000 rpm for 10 min at 10 °C. A 1500 μL of the supernatant was transferred to a clean vial and 200μL of the sample was injected into the SPE-UHPLC-MS/MS system for analysis.

### 3.5. Creatinine

The excretion amount of OXA and its main metabolite in urine were normalized to creatinine concentration. Creatinine analysis of the urine samples, based on the enzymatic procedure using a Dimension Vista 1500 system (Siemens Healthcare, Erlangen, Germany), was performed by the clinical laboratory SK-Lab (Lucenec, Slovakia). The principle of the enzymatic method is a reaction of peroxide with a chromogen in the presence of peroxidase enzyme to obtain a colored end product which is measured at 540 and 700 nm. The peroxide is created from creatinine which is hydrolyzed by creatininase and creatinase to sarcosine. Sarcosine oxidase hydrolyzes sarcosine to glycine, formaldehyde, and peroxide. The amount of peroxide is proportional to the concentration of creatinine in the sample. The analytical measurement range of the creatinine in the urine matrix was from 250 to 35,400 μM. The urine samples were measured directly from the specimen without any pretreatment (except dilution).

### 3.6. Method Validation

The SPE-UHPLC–MS/MS method was validated for the linearity, sensitivity, precision, recovery, accuracy, matrix effect, stability, the limit of quantification (LOQ), and limit of detection (LOD), according to the FDA guidelines (US Department of Health and Human Services Food and Drug Administration) [34].

Data from calibration (linearity, linear range, LOD, LOQ). The calibration was tested by using a series of the OXA standards in the concentration range of 20–5000 pg·mL^−1^ with the internal standard (1000 pg·mL^−1^) and applying a weighted (1/x) least-squares linear regression fit. Each calibration sample was measured 3 times. The calibration line was prepared in urine matrices and evaluated for the linearity (via determination coefficient) and linear range. The LOD and LOQ values were calculated from the calibration line, based on standard deviation of the response (SD_a_) and the slope (b):

LOD = 3 × SD_a_/b, (1)

LOQ = 10 × SD_a_/b, (2)

Data from QC (precision, accuracy, recovery, matrix effect, stability). The intraday precision and accuracy were calculated from 5 repeated injections at three concentration levels (100 pg·mL^−1^, 250 pg·mL^−1^, 1000 pg·mL^−1^) of OXA in the spiked model (blank) urine samples. The interday precision and accuracy of the method were determined by analyzing the spiked samples over 3 consecutive days. The analytical recovery was evaluated by comparing measured values of QC samples with three concentration levels of the OXA standards (75, 750, 5000 pg·mL^−1^) spiked into blank urine with corresponding nominal values (calculated from a calibration curve prepared in the same urine matrix). The matrix effect (ME) was calculated by the formula:

ME = [(A − B)/A] × 100,(3)

where A is the peak area of OXA standard in a mobile phase matrix and B is a peak area of the same OXA concentration spiked into a urine blank matrix. The autosampler stability was tested by analyzing the QC samples stored in the autosampler at 6 °C for up to 12 h. The freeze−thaw stability was tested by analyzing the QC samples after three freeze−thaw cycles at −70 °C. The results of the stability tests were calculated by comparing the concentrations after testing with those found in freshly prepared QC samples.

### 3.7. Drug Administration and Sample Collection

A 10 mg dose of OXA (Oxandrix) was administered orally to one healthy volunteer. Then, the urine samples were collected into 50 mL tubes after 4 h, 10 h, 20 h, 40 h, 48 h, 3.5 d, 4 d, 7 d, 8 d, 9 d, 10 d, 11 d, and 12 d from the oral administration. The urine samples were immediately stored at −20 °C for further analysis.

The subject gave his informed consent for inclusion before he participated in the study. This work and all its experiments, including the sample collection from humans, were approved by the Ethical Committee of Matej Bel University in Banska Bystrica, Slovakia.

## 4. Conclusions

The developed 2D LC-MS approach represents an attractive solution for the monitoring of trace OXA and its structurally related metabolite epi-oxandrolone in human urine matrices. The main benefits include (i) minimum sample handling and preparation (due to on-line SPE preconcentration and clean-up), (ii) short analysis time and high sample throughput (due to UHPLC), (iii) high sensitivity (due to QQQ detection), (iv) high selectivity (due to the 2D on-line SPE-UHPLC-MS/MS arrangement and given operating conditions). The on-line sample clean-up and subsequent liquid chromatography-tandem mass spectrometry analysis allowed the sample throughput to be 11 min per sample which represents ca. 65 samples analyzed within 12 h. Considering high reliability and a high degree of automation of the analytical process, the developed method is useful for routine use such as clinical or antidoping control.

## Figures and Tables

**Table 1:** The MS conditions for oxandrolone and methandienone in SRM mode.

Compound Name	Parent (m/z)	Daughter (m/z)	Dwell (s)	Cone (V)	Collision (V)
Oxandrolone	307.3	271.2 *	0.044	28	12
Oxandrolone	307.3	289.3	0.044	28	12
Oxandrolone	307.3	253.1	0.044	28	16
Oxandrolone	307.3	229.1	0.044	28	16
Oxandrolone	307.3	92.9	0.044	28	36
Metandienone (IS)	301.3	149.2	0.044	28	12

* used for quantification.

**Table 2:** Calibration and selected performance parameters of SPE-UHPLC-MS/MS (QQQ) method for oxandrolone.

Parameter	Oxandrolone
Linear range [pg·mL^−1^]	81.63–5000
R_t_ [min]	5.23
SD_Rt_ [min]	0.03
RSD_Rt_ [%]	0.57
W_1/2_ [min]	0.09
N	18730
H [mm]	0.00534
Slope (a)	0.000779
SD_a_	0.0000201
Intercept (b)	0.01517
SD_b_	0.00635
r^2^	0.99704
LOD [pg·mL^−1^]	24.49
LOQ [pg·mL^−1^]	81.63

R_t_—retention time; SD_Rt_—standard deviation of the retention time; RSD_Rt_—relative standard deviation of the retention time; w_1/2_—the peak width at half height; N—separation efficiency (number of theoretical plates; H—height equivalent to one theoretical plate; SD_a_—standard deviation of the slope; SD_b_—standard deviation of the intercept; r^2^—coefficient of determination; LOD—limit of detection; LOQ—limit of quantification.

**Table 3:** Intra- and Interday accuracy and precision.

	QC Level	Intraday (n = 5, Single Batch)			Interday (n = 15, 5 From Each Batch)		
Compound	Spiked Concentration [pg·mL^−1^]	Mean Concentration	RSD %	Accuracy %	Mean Concentration	RSD %	Accuracy %
**Oxandrolone**	100	109.5	3.26	109.5	106.5	10.98	106.5
	250	231.5	7.42	92.61	233.6	7.42	93.43
	1000	1012	2.38	101.2	1007	2.77	100.7

**Table 4:** Recovery and matrix effect.

Compound	Spiked Concentration [pg·mL^−1^]	Mean Concentration (n = 5)	Recovery % (n = 5)	Matrix Effect % (without IS), n = 5	Matrix Effect (with IS), n = 5
**Oxandrolone**	75	80.33	107.1	61.25	−8.63
	750	661.9	88.25	67.30	15.85
	5000	4870	97.39	56.64	−1.17

## Data Availability

The data is not available.

## Supplementary Materials

The following are available online, Table S1: Optimization of the MS conditions. Table S2: Optimization of UHPLC separation (stationary phase). Table S3: Optimization of UHPLC separation (mobile phase). Table S4: Optimization of the SPE procedure. Table S5: Stability of oxandrolone in urine matrix under different conditions. Table S6: Peak areas of oxandrolone in enzymatically hydrolyzed and nonhydrolyzed urine samples. Table S7: Concentration of oxandrolone in urine taken after administration of one dose (10 mg) of oxandrolone in tablet Oxandrix. Table S8: Gradient of mobile phase and positions of the switching valves in SPE enrichment process. Figure S1: Calibration curve of oxandrolone.
