# Supplementary material for: Fast and Sensitive Screening of Oxandrolone and Its Major Metabolite 17-Epi-Oxandrolone in Human Urine by UHPLC—MS/MS with On-Line SPE Sample Pretreatment
Source: Molecules. 2021 Jan 18;26(2):480. doi: 10.3390/molecules26020480 (PMC7831107; doi:10.3390/molecules26020480)
Supplement: Supplementary file 1 [file molecules-26-00480-s001.pdf]

molecules

Article

Fast and sensitive screening of oxandrolone and its major metabolite 17-epi-oxandrolone in human urine by UHPLC – MS/MS with on-line SPE sample pretreatment

Supplementary Material

Table of content

Table S1: Optimization of the MS conditions.
Table S2: Optimization of UHPLC separation (stationary phase).
Table S3: Optimization of UHPLC separation (mobile phase).
Table S4: Optimization of the SPE procedure.
Table S5: Stability of oxandrolone in urine matrix under different conditions.
Table S6: Peak areas of oxandrolone in enzymatically hydrolyzed and non-hydrolyzed urine samples.
Table S7: Concentration of oxandrolone in urine taken after administration of one dose (10mg) of oxandrolone in tablet Oxandrix®.
Table S8: Gradient of mobile phase and positions of the switching valves in SPE enrichment process.

Figure S1: Calibration curve of oxandrolone.

Table S1: Optimization of the MS conditions. The highlighted data (in bold) represent optimal values.

Cone voltage [V]		Desolvation gas flow [L.h^{-1}]		Desolvation gas temperature (°C) °C		Capillary voltage [kV]	
Tested range	*Intensity %*	*Tested range*	*Intensity %*	*Tested range*	*Intensity %*	*Tested range*	*Intensity %*
2	20	500	80	200	70	1.00	50.00
22	80	600	90	250	70	1.50	80.00
**28**	**100**	**700**	**100**	300	90	2.00	95.00
40	80	800	100	350	95	2.50	95
60	0	900	95	370	95	2.70	95
100	0	1000	95	**400**	**100**	**3.00**	**100**

molecules

MDPI

Table S2: Optimization of UHPLC separation (stationary phase).

Column type	Buffer	Temperature	% ACN	Retention time [min]	Width an half the height [min]	Column efficiency
Acquity UPLC BEH C18 (2.1X50, 1.7)	0.1% formic acid	40 °C	40	1.31	0.0411	5628
			50	0.79	0.0371	2512
			60	0.59	0.0355	1530
			70	0.50	0.0322	1336
			80	0.46	0.0298	1320
			90	0.42	0.0297	1108
Acquity UPLC BEH C8 (2.1X50, 1.7)	0.1% formic acid	40 °C	40	1.19	0.0387	5238
			50	0.75	0.0381	2147
			60	0.59	0.0306	2060
			70	0.52	0.0300	1664
			80	0.45	0.0292	1316
			90	0.45	0.0288	1353
Acquity UPLC CSH C18 (2.1 X50, 1.7)	0.1% formic acid	40 °C	40	1.55	0.0436	7002
			50	0.90	0.0411	2657
			60	0.66	0.0405	1471
			70	0.55	0.0305	1802
			80	0.49	0.0284	1649
			90	0.46	0.0357	920
Acquity UPLC HSS Cyano (2.1X50, 1.8)	0.1% formic acid	40 °C	40	1.09	0.0440	3400
			50	0.73	0.0372	2133
			60	0.58	0.0306	1990
			70	0.50	0.0285	1705
			80	0.45	0.0305	1206
			90	0.45	0.0269	1550
Acquity UPLC BEH Shield RP18 (2.1X50, 1.7)	0.1% formic acid	40 °C	40	1.40	0.0372	7847
			50	0.84	0.0315	3940

			60	0.63	0.0283	2745
			70	0.52	0.0275	1981
			80	0.45	0.0251	1781
			90	0.45	0.0263	1622
Acquity UPLC HSS T3 (2.1X50, 1.8)	0.1% formic acid	40 °C	40	2.04	0.0431	12411
			50	1.11	0.0343	5802
			60	0.77	0.0290	3906
			70	0.61	0.0289	2468
			80	0.52	0.0259	2233
			90	0.49	0.0261	1953

Table S3: Optimization of UHPLC separation (mobile phase).

Buffer	Temperature	% ACN	Retention time [min]	Width an half the height	Peak area	Column efficiency
0.1% formic acid	40 °C	50	1.11	0.0329	100026	6306
10 mM AF	40 °C	50	1.09	0.0405	70657	4013
20 mM AF	40 °C	50	1.09	0.0427	55592	3610

AF – ammonium formate

Table S4: Optimization of the SPE procedure.

Column type	Temperature	% ACN Load	Injection volume [µL]	Peak area – 2D	Recovery [%]
On-Line Extraction Column Xbridge C18 Direct Connect HP 10um (2.1X30)	40 °C	30	10	652562.6	92.36
				653831.8	92.54
		30	50	2987391	84.56
				3009281	85.18
		30	100	3581820	50.69
				3935258.7	55.70
		30	200	5372730	38.02
				5020543.05	35.53
On-Line Extraction Column Xbridge C8 Direct Connect HP 10um (2.1X30)	40 °C	30	10	650045.15	92.00
				663446.7	93.90
		30	50	2638867	74.70
				2886090	81.69
		30	100	1930680	27.32
				2350128	33.26
		30	200	2896020	20.49
				2976828.8	21.07
On-Line Extraction Column Oasis HLB Direct Connect HP 20um (2.1X30)	40 °C	30	10	627102.38	88.75
				670818.33	94.94
		30	50	2638867	74.70
				2886090	81.69
		30	100	1930680	27.32
				2350128	33.26
		30	200	2896020	20.49
				2976828.8	21.07

On-Line Extraction Column Acquity HSS T3 C18 Column (1.7 µm, 2.1 × 50 mm)	40 °C	30	10	726521.05	102.82
				700304.85	99.11
		30	50	3279735	92.84
				3386346	95.85
		30	100	6757380	95.64
				6834955.6	96.73
		30	200	12549420	88.81
				13121548	92.85

Table S5: Stability of oxandrolone in urine matrix under different conditions.

Conditions	Spiked concentration [pg.mL^{-1}]	Mean [pg.mL^{-1}]	Recovery %
**Autosampler stability at 6°C after 12h**	75	72.10	96.13
	750	669.6	89.28
	5000	4436	88.72
**Freeze–thaw cycle in urine (−70 °C, after the third cycle)**	75	68.05	90.73
	750	641.9	85.58
	5000	4436	88.72

Table S6: Peak areas of oxandrolone in enzymatically hydrolyzed and non-hydrolyzed urine samples.

	Area (sample1)		Area (sample2)	
**Sampling time**	**Hydrolyzed**	**Non-hydrolyzed**	**Hydrolyzed**	**Non-hydrolyzed**
10h after administration	1368.2	1451.0	11050	1052.0
20h after administration	1545.2	1559.9	1463.9	1566.0
48h after administration	124.98	118.15	178.50	150.20

molecules

Table S7: Concentration of oxandrolone in urine taken after administration of one dose (10mg) of oxandrolone in tablet Oxandrix®.

Time [hours]	c (OXA) Mean [pg.mL^{-1}]	SD [pg.mL^{-1}]	Creatinine [µmol.L^{-1}]	ng OXA /mmol creatinine	Area ratio Epi-oxandrolone/OXA
4	86137	9475	13900	6.2	0.124
10	151063	16617	18300	8.3	0.123
20	163903	18029	10900	15	0.117
40	6903	759.3	531.0	13	0.222
48	9081	998.9	3200	2.8	0.269
87.5	4235	465.9	11100	0.382	0.616
96	3150	346.5	5800	0.543	0.824
120	1418	155.9	8700	0.163	0.800
144	529.3	58.2	7200	0.074	0.835
168	329.3	36.2	16900	0.019	0.921
192	267.7	29.4	8200	0.033	-
216	99.3	10.9	8190	0.0121	-
240	42.6	4.7	11520	0.0037	-

molecules

Table S8: Gradient of mobile phase and positions of the switching valves in SPE enrichment process.

t [min]	B [%]	Flow [mL]	Left valve position	
0	30	0.4	Position 1	SPE column-waste
2.5	30	0.4		
2.6	30	0.4	Position 2	SPE-Analytical column to MS
5.5	90	0.4		
8.9	90	0.4		
9.0	30	0.4		
10	30	0.4	Position 1	SPE column-waste
11	30	0.4		

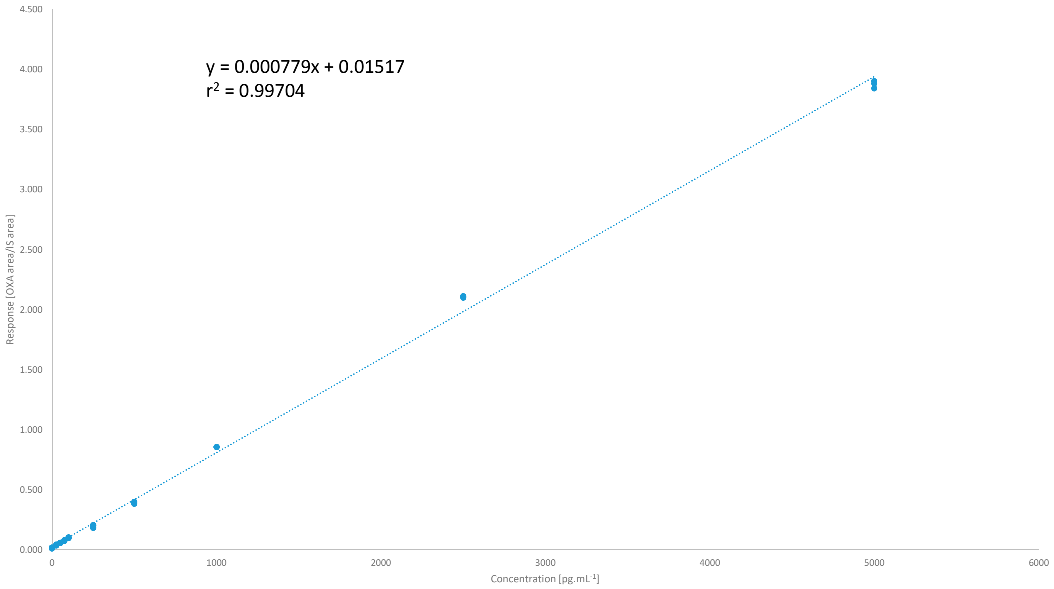

Figure S1: Calibration curve of oxandrolone.
